# Pro-Arrhythmic Effect of Chronic Stress-Associated Humoral Factors in Human Induced Pluripotent Stem Cell-Derived Cardiomyocytes

**DOI:** 10.3390/biology14060652

**Published:** 2025-06-04

**Authors:** Shi Su, Jinglei Sun, Suhua Qiu, Wenting Wu, Jiali Zhang, Yi Wang, Chenxia Shi, Yanfang Xu

**Affiliations:** 1 Department of Pharmacology, Hebei Medical University, Shijiazhuang 050017, China; sushiwudi@163.com (S.S.); 18712919298@163.com (J.S.); qiusuhua1111@hotmail.com (S.Q.); 15903213515@163.com (W.W.); ll13931860169@outlook.com (J.Z.); btwy001@163.com (Y.W.); chenxiashi@163.com (C.S.); 2 The Key Laboratory of New Drug Pharmacology and Toxicology of Hebei Province, Shijiazhuang 050017, China; 3 The Key Laboratory of Neural and Vascular Biology, Ministry of Education, Shijiazhuang 050017, China

**Keywords:** chronic stress, arrhythmia, potassium channels, human induced pluripotent stem cell-derived cardiomyocytes

## Abstract

Under chronic stress, the pro-arrhythmic effect and mechanism of circulating humoral factors in human cardiomyocytes remain unknown. In the present study, we observed the effect of serum from chronic-stress mice on the electrical activity of human-induced pluripotent stem cell-derived cardiomyocytes (hiPSC-CMs). Male C57/BL6J mice were subjected to 35 days of chronic unpredictable mild stress (CUMS). The serum from CUMS mice induced arrhythmia-like events (cell arrhythmias) in hiPSC-CMs in a time- and concentration-dependent manner. Patch clamp recordings in the heterologous expression system demonstrated that the serum derived from CUMS mice exerted an inhibitory effect on the cloned human potassium currents (*I*_to_, *I*_Kr_, *I*_Ks_) that mediate action potential repolarization. In addition, serum from CUMS reduced the expression of relevant channel proteins. Moreover, both heat-inactivated serum and deproteinized serum evoked similar severity of cell arrhythmias in hiPSC-CMs as the untreated serum, indicating that circulating substances with small molecules were mainly involved in the occurrence of arrhythmias. Furthermore, metabolomics analysis showed that 90 small-molecule metabolites increased and 390 decreased in CUMS serum. We concluded that circulating humoral substances under chronic stress conditions have direct arrhythmogenic effects by inducing ion channel dysfunction in myocardial cells.

## Simple Summary

The relationship between chronic stress and cardiovascular disease has been proven, but the mechanism is not clear at present. The study is devoted to analyzing the pro-arrhythmic effect of chronic unpredictable mild stress on human-induced pluripotent stem cell-derived cardiomyocytes and the underlying mechanisms. We employed the chronic, unpredictable mild stress model to simulate chronic stress. Through in vivo and in vitro electrocardiographic experiments, we demonstrated that chronic mild stress significantly increases arrhythmia susceptibility. Mouse serum, incubated with human-induced pluripotent stem cell-derived cardiomyocytes, was used to assess the pro-arrhythmic effects of humoral factors. Patch clamp and Western blot analyses revealed that decreased *I*_Kr_, *I*_Ks_, and *I*_to_ currents contribute to electrical remodeling, driven by the downregulation of ion channels. It was found that *I*_Ks_ is most sensitive to stress. Additionally, we conducted metabolic research to identify potential pro-arrhythmic metabolites. We discovered several serum metabolites that are associated with electrophysiological remodeling in human cardiomyocytes. This study evaluated the effect and mechanism of humoral factors on pro-arrhythmia. Our findings provide insights into the mechanisms underlying chronic stress-induced arrhythmias and may inspire novel preventive strategies for chronic stress-associated arrhythmias.

## 1. Introduction

The relation between chronic stress and cardiovascular disease has been proven [1,2,3,4]. Stress-induced malignant cardiac arrhythmias, such as ventricular tachycardia and ventricular fibrillation, are common contributors to sudden cardiac death (SCD) [5]. Understanding the mechanism of stress-induced electrophysiological remodeling that causes arrhythmia is a prerequisite for developing effective prevention strategies.

Neurological and humoral factors are the main causes of biological changes in cardiomyocytes leading to myocardial remodeling [6]. Neurohumoral factors are considered crucial in inducing arrhythmia. Sympathetic and parasympathetic systems are firstly activated by stress, and the excitability of parasympathetic nervous decrease follows [7]. Furthermore, the hypothalamic–pituitary–adrenal (HPA) axis may accelerate arrhythmia progress [8]. Humoral factors, such as catecholamine release and systemic inflammation, may increase cardiac repolarization abnormalities [9].

Life-threatening arrhythmias are often invoked by abnormal functions in ventricular repolarization and refractoriness [10,11], which themselves are often caused by a change in the outward K^+^ currents of cardiomyocytes. This dysfunction may be induced by heredity defects or acquired factors which can lead to electrical remodeling [12].

The action potential (AP) of cardiomyocytes is regulated by transmembrane currents mediated by multiple ion channels, and the dysfunction of these channels presents a molecular mechanism of cardiac arrhythmia generation. Chronic stress has been shown to induce ventricular repolarization-related ion remodeling in rodent models of chronic unpredictable mild stress (CUMS), such as in rats and mice [13,14]. It has been reported that in CUMS models, the transient outward potassium current (*I*_to_) and L-type calcium current (*I*_Ca-L_) decrease [13,14,15]. However, due to the limitation of animal models, previous studies have primarily focused on rodents when researching stress-related arrhythmia. Unlike in small rodents, ventricular repolarization in the human heart is mainly mediated by the slowly (*I*_Ks_) and rapidly delayed rectifier potassium currents (*I*_Kr_) [16]. Currently, there is a lack of research on human cardiac electrical activity under stress. This gap severely hinders our in-depth understanding of the pathophysiological mechanisms of stress-related arrhythmias in humans and the development of targeted therapeutic strategies.

Human-induced pluripotent stem cell-derived cardiomyocytes (hiPSC-CMs) are derived from induced pluripotent stem cells (iPSC), which are generated by reprogramming somatic cells. The hiPSC-CMs exhibit high similarity to human primary cardiomyocytes in electrophysiological properties and drug reactivity. They are a valuable tool in basic research on the effect of stress on cardiomyocytes at the human level [17]. It has been demonstrated that the humoral environment could be simulated by incubating hiPSC-CMs with serum from patients [18].

In this study, we incubated hiPSC-CMs with the serum from mice in the CUMS model to mimic the humoral environment of chronic stress and assessed the effect of humoral factors on the electrical activity of cardiomyocytes. Additionally, we detected the changes in key currents mediating the repolarization of cardiac AP and the expression of corresponding ion channels. Finally, metabolomics analysis was conducted to identify the differential metabolites that may contribute to arrhythmia.

## 2. Materials and Methods

### 2.1. Animals

We purchased 8-week-old male C57/BL6J mice from Beijing Vital River Laboratory Animal Technology Co., Ltd., Beijing, China. The animals were housed under standard conditions (25 ± 2 °C, 50–60% humidity, 12 h/12 h light–dark cycle) with one week of habituation, with food and water provided ad libitum. Animals were randomly grouped using a random-number method, with no more than five mice in each cage. This research was conducted in accordance with the guidelines of the Animal Care and Use Committee of Hebei Medical University and approved by the Animal Ethics Committee of Hebei Medical University (approval number: 2017013).

### 2.2. Procedure of CUMS

The C57/BL6J mice were randomly divided into control and CUMS groups. The CUMS procedure and behavioral tests, including the sucrose preference test (SPT), open field test (OFT), elevated plus maze (EPM) test, tail suspension test (TST), and forced-swimming test (FST), were performed as described previously [19,20,21,22,23]. During CUMS model establishment, each mouse was housed in an individual cage. Detailed information about the CUMS and behavioral tests can be found in the Supplementary Files. The number of mice was sufficient to elucidate the difference between the two groups. Mice in CUMS group that satisfied the requirements of at least three behavioral tests were selected for subsequent experiments. The number of animals in each experiment is indicated in the corresponding figure legends.

### 2.3. Electrocardiographic Recordings

The mice were anesthetized with isoflurane at a dose of 2–3% for induction and 1–1.5% for maintenance. Body surface electrocardiograms (ECGs) were recorded using the Biopac150 System (Biopac Systems, San Jose, CA, USA) with standard lead II. To evaluate the mice’s susceptibility to arrhythmias, we administered isoproterenol (ISO) and caffeine and subsequently observed the changes in body surface ECG. To eliminate the effects of neurohumoral factors and metabolic processes, we further investigated the arrhythmia susceptibility of isolated hearts. Briefly, isolated hearts were perfused with oxygenated Tyrode’s solution and allowed to equilibrate in a thermostatic chamber for a minimum of 30 min. Subsequently, Tyrode’s solution containing ISO (1 μM) was perfused. The in vitro equivalent to lead II ECG waveforms were obtained continuously throughout the entire perfusion process. The PR, QT, and RR intervals were measured directly, as reported previously, and Bazett’s equation QTc = QT/(RR^0.5^) was applied for the corrected QT interval (QTc) calculation [24,25].

### 2.4. Serum Preparation and Treatment

Blood samples from 3 batches of CUMS mice were obtained by abdominal aortic puncture from control and CUMS mice, and serum was isolated by centrifugation at 4 °C and then stored at −80 °C. Inactivated serum was prepared by heating at 56 °C for 30 min. Serum extracts were prepared by precipitating proteins from fresh serum with methanol. After centrifugation, the supernatant was collected and freeze-dried under N_2_.

### 2.5. Culturing of hiPSC-CMs and Monitoring Using CardioExcyte96

Commercially available hiPSC-CMs (purchased from Beijing Cellapy Biotechnology Co., Ltd., Beijing, Chnia, catalog number: CA2201106) were used to record the electroactivity of cardiomyocytes. The hiPSC-CMs predominantly consisted of ventricular myocyte-like cells with a small population of atrial myocyte-like cells, possessing characteristics similar to human cardiomyocytes, such as electrophysiological properties and contraction ability [26,27,28]. The hiPSC-CMs were generated from iPS cells derived from a 55-year-old healthy female donor. The iPS cells underwent a specific differentiation protocol to obtain hiPSC-CMs, which were subsequently stored in liquid nitrogen (handled by the supplier). Cardiomyocytes were identified by using cardiomyocyte-specific protein antibodies (cardiac troponin T and α-actinin). The hiPSC-CMs were thawed and cultured according to the manufacturer’s instructions. The hiPSC-CMs were diluted in pre-warmed plating medium (Beijing Cellapy Biotechnology, catalog number: CA2013100) and plated in the wells of 0.6 mm NSP-96 CardioExcyte 96 sensor plates (Nanion, Munich, Germany) pre-coated with Matrigel for extracellular field potential (EFP) recording. The humidified incubator was used for cardiomyocyte cultivation at 37 °C in 5% CO_2_ for 48 h. The cells were then washed to remove debris, and the test serum was added 4 h after the medium was replaced by a maintenance medium.

The NSP-96 CardioExcyte 96 sensor plate is a specialized plate featuring planar gold electrodes embedded at the bottom. It enables long-term, non-invasive, and label-free acquisition of electrical activity (via EFP) and contraction (via impedance) from the entire cell population through the CardioExcyte 96 system [29]. NSP-96 CardioExcyte 96 sensor plates with 6 mm electrode size are more favorable for EFP recording. Serum from either control or CUMS mice was added at different final concentrations (V/V) for a 48 h period, and EFP was analyzed using CardioExcyte Control software. We used the serum from three batches of CUMS mice models for investigation of pro-arrhythmic effect on hiPSC-CMs. We defined arrhythmia-like events as “cell arrhythmias” in EFP waveforms according to previous studies [30]. The cell arrhythmia of hiPSC-CMs was observed and categorized from mild to serious, as reported previously for the categories normal, early afterdepolarization (EAD), rolling EAD, ectopic beat, irregular beat, and tachycardia, with scores ranging from 1 to 6 [31].

### 2.6. Cell Culture

We applied human embryonic kidney 293 cells (HEK 293 cells) obtained and produced from Inovogen Tech. Co. (Beijing, China), which stably expressed K_V_4.3/KChIP2 (for *I*_to_ recording), KCNQ1/KCNE1 (for *I*_Ks_ recording), or hERG (for *I*_Kr_ recording). The cells were cultured in a humidified incubator with 5% CO_2_ at 37 °C in Dulbecco’s Modified Eagle’s Medium (DMEM, Gibco, Grand Island, NY, USA) mixed with 10% fetal bovine serum (Excell, Suzhou, China) and 1% penicillin/streptomycin solution (Gibco, Grand Island, NY, USA).

### 2.7. Patch-Clamp Recordings

Whole-cell current recordings were performed using a patch-clamp setup (HEKA EPC9, Reutlingen , Germany) to record cloned human *I*_Kr_, *I*_Ks_, and *I*_to_ in HEK 293 cells stably expressing these ion channels. Information about the external and pipette solutions was recorded in the Supplementary Files. Borosilicate glass electrodes with a tip resistance of 2–3 MΩ, when filled with the pipette solution, were used. Tail current densities (pA/pF) were obtained by normalizing tail current amplitudes by cell membrane capacitance. An axon patch 700B amplifier was used for relative experiments performed using pClamp 10.2 software (Molecular Devices, San Jose, CA, USA). Sampling was conducted at 2.5–10 kHz, and a low-pass filter was used at 1 kHz, while an A/D converter (Digidata 1322; Molecular Devices, San Jose, CA, USA) was used for digitization.

### 2.8. Western Blot Analysis

Protein was extracted from hERG-HEK293 cells, KCNQ1/KCNE1-HEK293 cells, and K_V_4.3/KChIP2-HEK293 cells, which had been treated with serum from either control or CUMS mice. The RIPA lysis buffer with 1% protease inhibitors was applied for protein extraction, while protein concentration was determined using the BCA assay (Multi science, Hangzhou, China). Total protein was separated using precast SDS-PAGE gel (ACE, Changzhou, China) and transferred onto PVDF membranes. The Odyssey Infrared Imaging System (Li-COR, Lincoln, NE, USA) was used for quantification. Anti-K_V_4.3 (Alomone, Jerusalem, Israel), anti-K_V_11.1 (Alomone), anti-K_V_7.1 (Alomone), anti-GAPDH (Proteintech, Rosemont, IL, USA), and anti-Na^+^-K^+^-ATPase (Millipore, Billerica, MA, USA) were applied as primary antibodies.

### 2.9. LC-MS/MS Analysis and Metabolomics

The metabolomics was accessed by the method of LC-MS/MS. The ChemSpider, mzCloud, and Mass List Match databases were used to identify and match the metabolites. The obtained data matrix was uploaded to the Weishengxin platform (https://www.bioinformatics.com.cn/ (accessed on 21 April 2025)) for further analysis. After normalization, the principal component analysis (PCA) method was applied to test the difference between the CUMS and control groups, and metabolites with |log_2_foldchange| ≥ 1.0 and *P*_adj_ ≤ 0.05 were determined as significant.

### 2.10. Statistical Analysis

The data were presented as the mean ± SEM, and their analysis was conducted by staff who were blinded to the grouping details. Statistical analysis was performed with SPSS 27 (SPSS, Chicago, MI, USA) using unpaired Student’s *t*-tests (for two-group comparisons), paired Student’s *t*-tests (for comparisons of before and after treatment), or Mann–Whitney U tests, while the data did not conform to normality. *p* values of < 0.05 were considered statistically significant.

## 3. Results

### 3.1. The CUMS Mice Had an Increased Susceptibility to Arrhythmia

**Figure 1:**
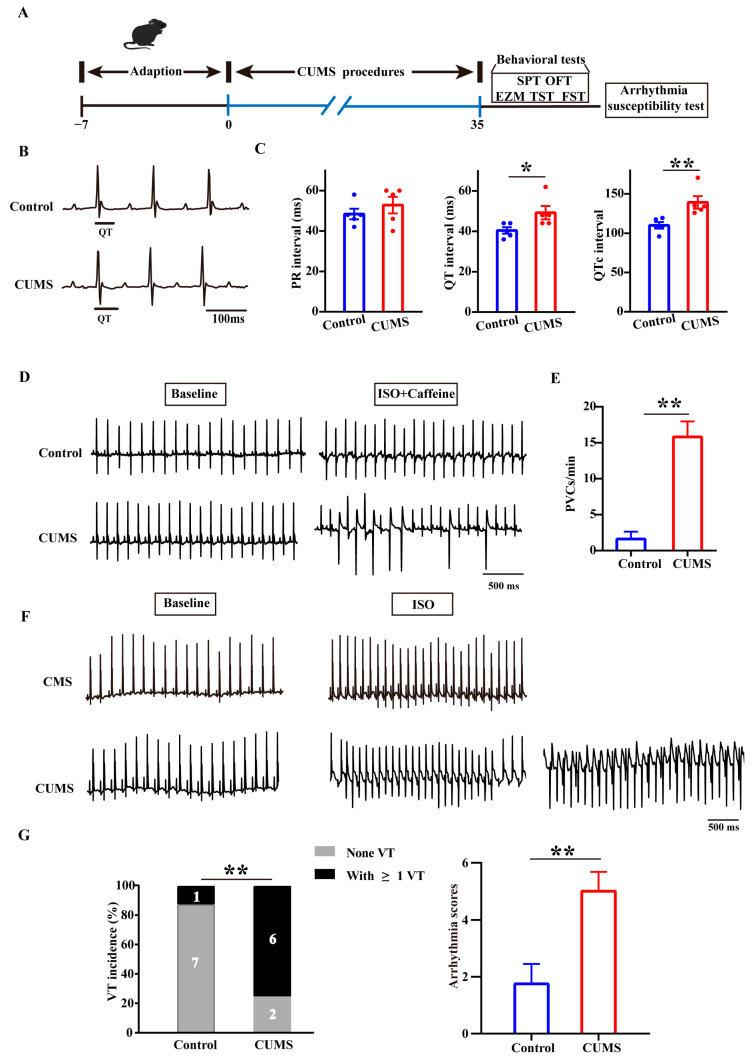
The CUMS increases the susceptibility of mice to arrhythmias. (**A**) The schematic schedule for the procedure of CUMS in the mouse model. (**B**) Typical traces of surface II lead ECG in control and CUMS group mice. (**C**) Summary data for ECG relative parameters, including PR interval, QT interval, and QTc. *n* = 5 in each group. (**D**) Representative traces of surface II lead ECG in mice treated with ISO and caffeine. (**E**) The number of PVCs occurring within one minute in surface ECG recording. PVCs were counted for the first 5 min after the initial PVC. *n* = 5 in each group. (**F**) Representative traces of ECG in vitro by Langendorff perfusion after treated with ISO. (**G**) The number of VT events and arrhythmia score. *n* = 8 in each group. Mean ± SEM. * *p* < 0.05, ** *p* < 0.01 vs. control group.

The establishment of the CUMS model in mice and the behavioral assessments were performed, as shown in Figure 1A. We dynamically assessed the body weights of the mice during the CUMS procedure and observed that CUMS significantly inhibited body weight gain (Figure S1A). Behavioral tests conducted following CUMS modeling revealed that these mice exhibited a decreased path distance in the center of the OFT, increased time in the open arms of the EPM, reduced preference for sucrose, and increased time of immobility in the TST and FST (Figure S1B–H). Subsequently, we assessed the cardiac electrical activity in the mice. In a resting state, the QT interval and QTc interval were significantly prolonged in the CUMS mice (Figure 1B,C). Under the induction of isoproterenol (ISO) and caffeine, the incidence of premature ventricular contractions (PVCs) was significantly higher in the CUMS mice than in the control mice (Figure 1D,E). Additionally, to minimize the effects of neural regulation on cardiac electrical activity, we performed isolated heart perfusion and administered ISO stimulation to investigate susceptibility to arrhythmias in the CUMS mice and found that they exhibited a higher frequency of VTs (Figure 1F,G). We integrated the Faggioni and van der Werf et al. scoring systems to grade ventricular arrhythmias of varying severity (PVC, 1; Bigemini, 2; Couplet, 3; Triplet, 4; VT < 1 s, 5; VT ≥ 1 s, 6) [32,33]; This revealed higher scores in the CUMS mice (Figure 1G). Overall, these results indicated that stress increases susceptibility to arrhythmias and that this effect is not attributable to direct neural regulation.

### 3.2. Pro-Arrhythmic Effect of CUMS Serum on Human Cardiomyocytes

**Figure 2:**
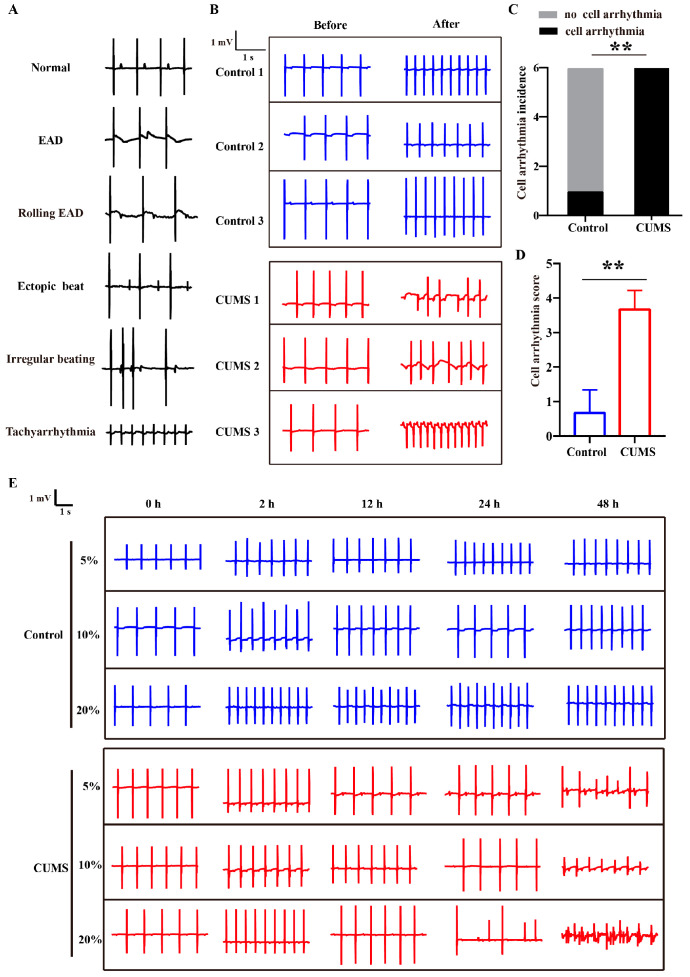

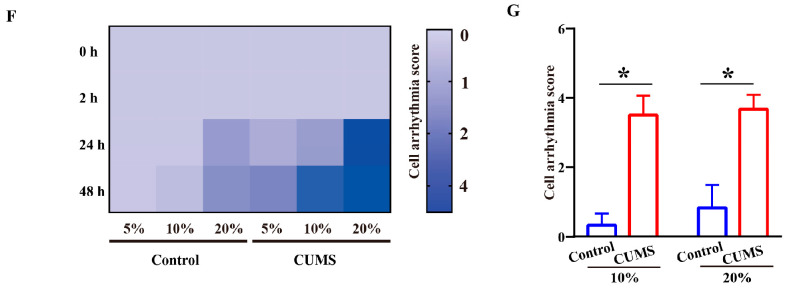
The CUMS mice serum induces arrhythmia in hiPSC-CMs. (**A**) Representative cell arrhythmia types recorded in hiPSC-CMs. (**B**) Representative EFP traces of cell arrhythmia events induced by CUMS mice serum. (**C**) Summary results of cell arrhythmia incidence. *n* = 6 in each group. (**D**) Summary results of cell arrhythmia scores. *n* = 6 in each group. (**E**) Representative EFP traces of cell arrhythmia induced by different concentrations of mice serum. (**F**) Heatmap of arrhythmia scores under different serum concentrations and incubation times. (**G**) Cell arrhythmia scores induced by 10% and 20% serum. *n* = 6 in each group. Mean ± SEM. * *p* < 0.05, ** *p* < 0.01 vs. control group.

Isolated heart perfusion demonstrated that CUMS mice exhibited increased susceptibility to arrhythmias, suggesting that the pro-arrhythmic effect is not attributable to direct neural regulation. Instead, it may be attributed to humoral factors associated with the stress state, which could lead to electrophysiological remodeling in mice hearts. To evaluate the influence of humoral factors of stress on the electrical activity of human cardiomyocytes, we isolated serum from CUMS mice and incubated it with hiPSC-CMs to observe changes in the electrical activity of hiPSC-CMs. The hiPSC-CMs were identified by cardiomyocyte-specific antibodies (α-actinin and cardiac troponin T [cTnT]) (Figure S2A). Serum from control mice was used as a comparison for synchronous observation. Figure S2B showed the morphology of hiPSC-CMs after treatment with serum from control mice or CUMS mice for 48 h. It was indicated that a portion of hiPSC-CMs treated with serum from CUMS mice displayed shrinkage. Further, according to the assessment of drug-induced arrhythmias based on hiPSC-CMs assays, the cell arrhythmias observed were defined and classified as EAD, rolling EAD, ectopic beat, irregular beat, and tachyarrhythmia (Figure 2A) [30,31]. Using a scoring system analogous to the ECG arrhythmia criteria, scores of 1 to 5 were assigned to these cell arrhythmia types to evaluate severity. The hiPSC-CMs incubated with CUMS mice serum exhibited various cell arrhythmia with higher incidence and arrhythmia scores, whereas control mice serum increased beating rate without inducing arrhythmia (Figure 2B–D). We further observed that the cell arrhythmias induced by serum from CUMS mice exhibited both serum dose-dependence and incubation time-dependence (Figure 2E,F). At the same serum concentration, serum derived from CUMS mice was more prone to inducing cell arrhythmia (Figure 2G). Additionally, we observed that CUMS mice serum typically induced cell arrhythmias after incubation for 24 and 48 h, suggesting the involvement of chronic regulation mechanisms. Collectively, these findings indicated that CUMS serum led to severe cellular arrhythmia in hiPSC-CMs.

### 3.3. The CUMS Serum Induced Dysfunction of Cloned Human Potassium Ion Channels

**Figure 3:**
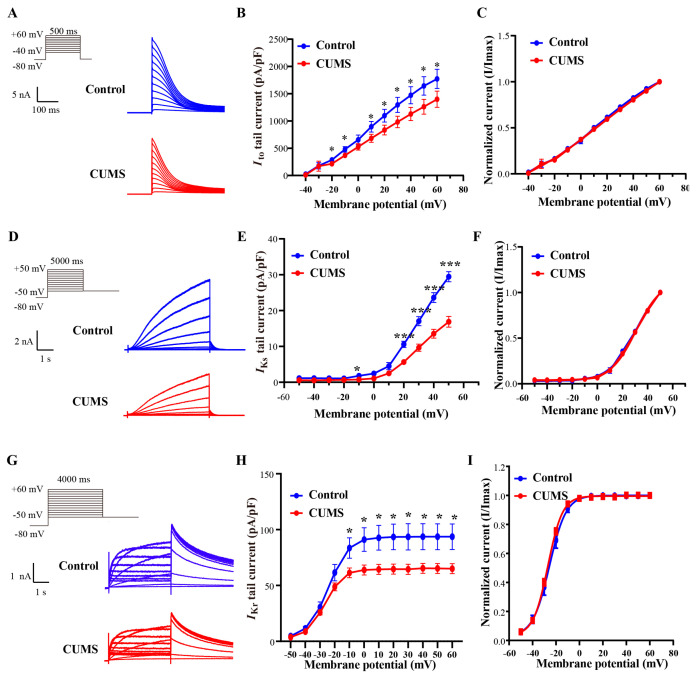
Chronic effect of serum from mice with CUMS on repolarization currents. (**A**) Representative *I*_to_ traces recorded in the presence of control serum and CUMS serum for 48 h. (**B**) Current density–voltage relationship of *I*_to_. *n* = 13 in control group, *n* = 10 in CUMS group. (**C**) Activation curve of *I*_to_ and the data fitted to Boltzmann equations. *n* = 13 in control group, *n* = 10 in CUMS group. (**D**) Representative *I*_Ks_ traces recorded in the presence of control serum and CUMS serum for 48 h. (**E**) Current density–voltage relationship of *I*_Ks_. *n* = 11 in each group. (**F**) Activation curve of *I*_Ks_ and the data fitted to Boltzmann equations. *n* = 11 in each group. (**G**) Representative *I*_Kr_ traces recorded in the presence of control serum and CUMS serum for 48 h. (**H**) Current density–voltage relationship of *I*_Kr_. *n* = 15 in each group. (**I**) Activation curve of *I*_Kr_ and the data fitted to Boltzmann equations. *n* = 15 in each group. Mean ± SEM. * *p* < 0.05, *** *p* < 0.001 vs. control group.

To investigate the mechanisms underlying the pro-arrhythmic effect of CUMS serum, we recorded the repolarizing potassium channel currents involved in the AP, namely *I*_to_, *I*_Kr_, and *I*_Ks_, in HEK cells stably expressing K_V_4.3/KChIP2, *hERG*, and KCNQ1/KCNE1, respectively. Considering that 10% and 20% CUMS serum exhibited similar pro-arrhythmic effects, we chose to employ 10% serum for further study. Since CUMS serum-induced cell arrhythmia in hiPSC-CMs after chronic incubation, we observed changes in various repolarization currents following 48 h incubation with CUMS serum. Figure 3A presented the representative traces of *I*_to_, the major current mediating the phase 1 repolarization of the AP, after incubation with control or CUMS serum. As depicted in Figure 3B, CUMS serum reduced *I*_to_ current densities across the voltage range of −20 to +60 mV, with a 24.1% reduction at +60 mV (1394.4 ± 103.6 pA/pF vs. 1836.1 ± 156.0 pA/pF). When fitting the activation curves with the Boltzmann function, we found that CUMS serum did not alter the activation of *I*_to_ (15.5 ± 4.3 mV vs. 13.5 ± 3.4 mV, *p* > 0.05) (Figure 3C). In addition, *I*_Ks_, a crucial current for repolarization reserve, showed a significant decrease across the voltage range of +20 to +50 mV after incubation with CUMS serum, with a 42.5% reduction in the maximal current densities of *I*_Ks_ at +50 mV (16.9 ± 1.5 pA/pF vs. 29.4 ± 1.4 pA/pF) (Figure 3D,E). Similarly, CUMS serum did not affect the steady-state activation of *I*_Ks_ (31.6 ± 1.6 mV vs. 31.9 ± 1.4 mV, *p* > 0.05) (Figure 3F). Moreover, the CUMS serum also caused a decrease of approximately 30.5% in the maximal current densities of *I*_Kr_ at +60 mV (65.1 ± 4.4 pA/pF vs. 93.7 ± 11.4 pA/pF), an important repolarization current in phase 3 of the ventricular myocyte AP (Figure 3G,H). And likewise, the CUMS serum did not alter the steady-state activation of *I*_kr_ (−26.9 ± 0.7 mV vs. −25.6 ± 1.7 mV, *p* > 0.05) (Figure 3I). We also observed that acute perfusion with 10% control or CUMS serum did not alter current densities (Figure S3A–F). This finding indicated that CUMS serum inhibits the repolarization of potassium currents through long-lasting mechanisms, thereby triggering arrhythmias.

### 3.4. The CUMS Serum Downregulated the Expression of Potassium Channels

**Figure 4:**
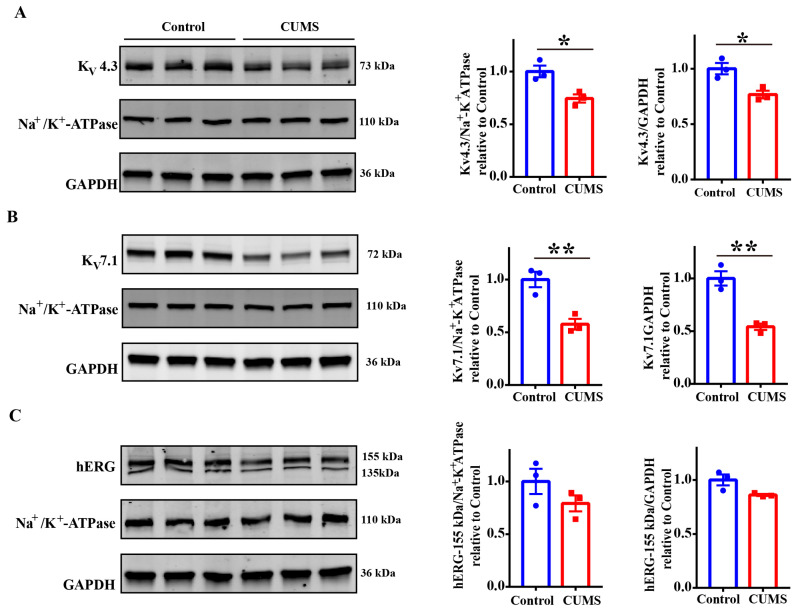
The effect of CUMS serum on the expression of ion channel proteins. (**A**) The expression of K_V_4.3 protein in K_V_4.3/KChIP2-HEK cells after treatment with control or CUMS serum for 48 h. *n* = 3 in each group. (**B**) The expression of KCNQ1 (K_V_7.1) in KCNQ1/E1 cells after treatment with control or CUMS serum for 48 h. *n* = 3 in each group. (**C**) The expression of hERG in hERG-HEK cells after treatment with control or CUMS serum for 48 h. *n* = 3 in each group. Mean ± SEM. * *p* < 0.05, ** *p* < 0.01 vs. control group. Original western blot figures in Figure S4.

Given that the long-term action of CUMS serum did not alter the current kinetics, we investigated whether CUMS serum achieved a reduction in the current by affecting the expression of channel proteins. Therefore, we detected the expression of various channel proteins after 48 h of incubation with CUMS serum. We found that CUMS serum reduced K_V_4.3 protein expression (mediating *I*_to_) by nearly 25% (Figure 4A). The CUMS serum also caused a more than 40% decrease in the expression of the K_V_7.1 protein, which mediates *I*_Ks_ (Figure 4B). As for the hERG protein, the mature form (155 kDa), which is located on the cell membrane and mediates *I*_Kr_, showed a trend of reduction in expression, but this trend was not statistically significant (Figure 4C). The reduction in channel proteins correlated well with the decrease in corresponding currents. Specifically, the K_V_7.1 protein exhibited the most profound protein decline, paralleling the largest current reduction in *I*_Ks_. K_V_4.3 (*I*_to_) followed this with a moderate decrease, and the smallest reduction was found in hERG (*I*_Kr_). These results indicated that CUMS serum downregulated the expression of potassium channel proteins mediating AP repolarization, thereby reducing repolarizing currents and promoting arrhythmia in cardiomyocytes.

### 3.5. Small-Molecule Metabolites Mainly Mediated the Pro-Arrhythmic Effects Caused by CUMS

**Figure 5:**
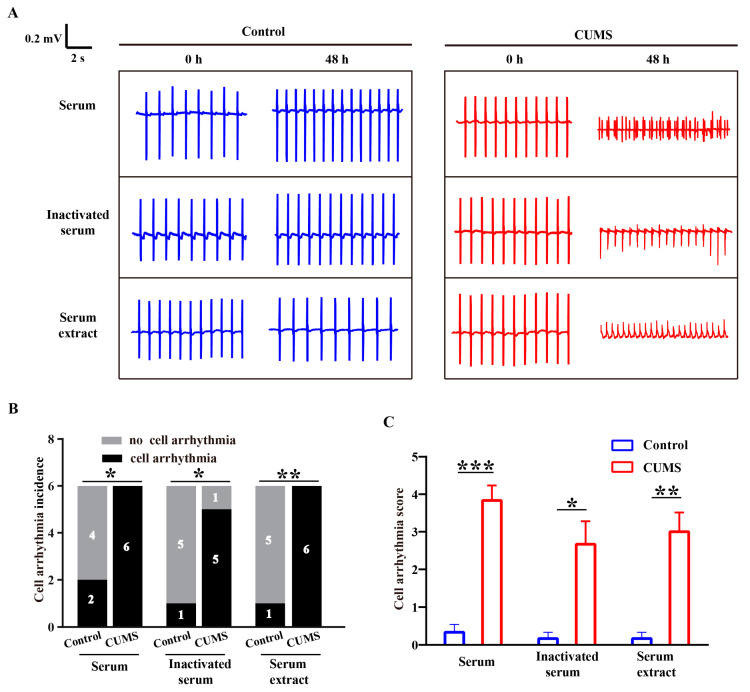
The effects of serum with different treatments on the EFP of hiPSC-CMs. (**A**) Representative EFP traces of hiPSC-CMs after incubation with serum, inactivated serum, and serum extract of mice. (**B**) Summary results of cell arrhythmia incidence. *n* = 6 in each group. (**C**) Summary results of cell arrhythmia scores. *n* = 6 in each group. Mean ± SEM. * *p* < 0.05, ** *p* < 0.01, *** *p* < 0.01 vs. control group.

It is known that serum contains various bioactive substances, including macromolecular proteins and small-molecule metabolites. To explore which factors in the serum are involved in the pro-arrhythmic effect caused by CUMS, we processed the serum into inactivated serum and serum extracts. Inactivated serum was obtained by subjecting fresh serum to a 30 min water bath at 56 °C, and serum extracts were prepared by precipitating proteins from fresh serum with methanol. We incubated hiPSC-CMs with serum, inactivated serum, and serum extracts at an equivalent dosage to the serum, observing changes in EFP. We found that both CUMS inactivated serum and CUMS serum extracts induced arrhythmias in hiPSC-CMs, comparably to native CUMS serum (Figure 5A). The incidence and severity scores of cell arrhythmias induced by CUMS-derived serum, inactivated serum, and serum extract were higher than those of control serum (Figure 5B,C). Our findings revealed that the small-molecule metabolites in CUMS serum possess intrinsic arrhythmogenic properties.

### 3.6. Analysis of Differential Metabolites in Serum

**Figure 6:**
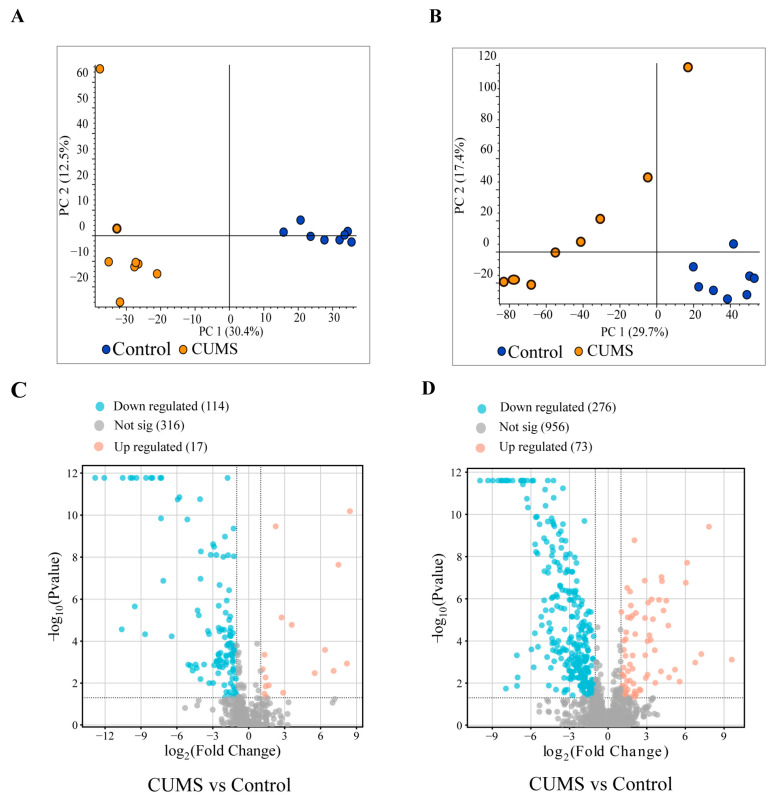
Metabolomics identification of metabolites in the serum of control and CUMS mice. (**A**) PCA score plot in negative ion mode. *n* = 8 in each group. (**B**) The PCA score plot in positive ion mode. *n* = 8 in each group. (**C**) Volcano plots of differential metabolites in negative ion mode. *n* = 8 in each group. (**D**) Volcano plots of differential metabolites in positive ion mode. *n* = 8 in each group.

To identify the differential small-molecule metabolites in the serum between the Control group and the CUMS group, we conducted non-targeted metabolomics on serum extracts. Principal component analysis (PCA) revealed distinct clustering of the samples from the two groups in both negative and positive ion modes, indicating significant differences between the control and CUMS groups (Figure 6A,B). The differential levels of small-molecule metabolites were presented through a volcano plot. Using the criteria of |log_2_foldchange| ≥ 1.0 and *P*_adj_ ≤ 0.05, we found that 17 substances were upregulated and 114 substances were significantly downregulated in the negative ion mode, while 73 substances were upregulated and 276 were significantly downregulated in the positive ion mode in the CUMS group (Figure 6C,D). Partial differential metabolites identified in CUMS mice serum are listed in Table S1. These small-molecule metabolites may induce arrhythmia by down-regulating channel expression and reducing ionic currents.

## 4. Discussion

In the present research, we investigated the electrophysiological mechanisms of arrhythmia induced by humoral factors under chronic stress. Humoral factors under stress were first proven to be critical to the electrophysiological remodeling of human cardiomyocytes with pro-arrhythmic effects. Serum from CUMS mice was used to explore the effects of humoral factors on electrical activity of hiPSC-CMs. We recorded the currents of ion channels that constitute the action potential to elucidate the mechanism underlying stress-induced arrhythmia. Additionally, metabolites in the serum were confirmed to play a critical role in human arrhythmogenesis.

In this study, the C57/BL6J mice were subjected to the CUMS procedure and exhibited obvious influence of stress, as evidenced by significant effects like changes in body weight and behavior, which was consistent with previous studies [22,34]. We selected CUMS as the method for inducing stress because unpredictable stressors could induce stronger behavioral and physiological changes compared to predictable stressors, similar to the multifactorial stressors encountered in modern life [35]. The C57/BL6J mice undergoing CUMS showed significant prolongation of QTc intervals, as determined by lead II ECG waveforms from electrocardiographs, and increased vulnerability to ventricular arrhythmias, as demonstrated by Iso stimulation.

Subsequently, we evaluated the electrical remodeling of human cardiomyocytes under stress conditions. We found that, when incubated with hiPSC-CMs for 48 h, serum from CUMS mice could induce cell arrhythmia in hiPSC-CMs in a dose-dependent manner. This indicated that humoral factors under stress are one of the common causes of human cardiomyocyte electrophysiological remodeling. Moreover, this study provided novel evidence of the pro-arrhythmic effects of stress-induced humoral factors by using hiPSC-CMs. It has been proven that the humoral environment could be simulated by incubating hiPSC-CMs with serum derived from patients [18]. In the present study, serum from mice was used to simulate humoral factors. We demonstrated that humoral factors under stress could induce changes in the FP of hiPSC-CMs, leading to the occurrence of cell arrhythmias. It was also illustrated that serum from CUMS mice has no acute effect on such occurrence, as shown by the results of 5 min perfusion, suggesting that the electrophysiological remodeling of human cardiomyocytes was induced by stressors via chronic effects, according to the dose- and time-dependent effects.

Based on the findings described above, stress was verified to affect electrophysiological remodeling, which increases the risk of susceptibility to arrhythmia or the occurrence of arrhythmia. We explored the mechanism of electrophysiological remodeling using a human ion channel expression system incubated with serum under stress for 48 h. The current densities of *I*_to_, *I*_Ks_, and *I*_Kr_ detected in our study showed a tendency for decreasing. It was revealed that *I*_Ks_ is the most sensitive current when exposed to humoral factors under stress. Previous research on stress has primarily focused on rodents. The prolongation of the QTc was observed in the mice receiving chronic stress. For further study, prolongation of APD at 90% repolarization was observed in CUMS group rats [15,36].

Increased susceptibility to ventricular arrhythmias was proven in rats under CUMS conditions. Rats in a model group undergoing CUMS showed lower *I*_to_ and *I*_Ca-L_ current densities than rats in a control group [15,36], and it was observed that the expression of K_V_4.2, K_V_4.3, and Ca_V_1.2 in cardiomyocytes was reduced [15]. Repolarization impairment is usually induced by the dysfunction of outward K^+^ currents, mainly including *I*_to_, *I*_Ks_, and *I*_Kr_ in human cardiomyocytes. Repolarization reserve is related to the redundancy of repolarizing currents, while normal repolarization can be maintained by the remaining currents when one of them is restrained. [37,38]. In other words, the relative status of each repolarizing current is related to the function and state of the other K^+^ channels. For instance, *I*_Ks_ is considered to have a minor role in usual conditions for repolarization in humans [39,40,41]. The suppression of *I*_Ks_ only is indicated to be a rare occurrence for APD prolongation in animals and human ventricular myocytes [42,43,44,45,46]. Accordingly, *I*_Ks_ is thought to be more important and to play a role in negative feedback to reduce the risk of arrhythmia genesis from delayed ventricular repolarization [44]. In our study, chronic stress led to the reduction in *I*_Kr_ and *I*_to_; meanwhile, we recorded a decrease in *I*_Ks_ decompensating repolarization reserve. We conducted the patch-clamp experiments on HEK cells. It would be useful to verify our findings on *I*_to_, *I*_Ks_, and *I*_Kr_ decreasing in hiPSC-CMs by better simulating the situation in the human heart for further study.

According to the findings above, serum stress could induce electrical remodeling. We can infer that active substances in the serum under stress induce remodeling in multiple ion channels. Humoral factors, combined with neurological factors, are mainly responsible for myocardial remodeling [6]. Among humoral factors, active substances in the renin–angiotensin–aldosterone system, adrenaline and norepinephrine, vasopressin, cytokines, and cortisol, which are identified as proteins, polypeptides, and small-molecule metabolites, are related to electrical remodeling [47,48,49,50,51].

To investigate the effects of metabolites in stress serum, inactivated serum and extracts of serum metabolites were obtained to eliminate the impact of active proteins. Both inactivated serum and extracts of serum metabolites were administered for 48 h incubation with hiPSC-CMs. Both inactivated serum and serum metabolite extracts exhibited a pro-arrhythmic effect, indicating that the metabolites in serum can cause electrical remodeling in cardiomyocytes through chronic effects. In our study, it was revealed that a number of metabolites, such as adenine and adenosine, increased, while 5α-dihydrotestosteronein (DHT), docosahexaenoic acid (DHA) decreased in the serum of CUMS group. It is suggested that the combined effect of these changed metabolites may contribute to pro-arrhythmia. As for the single metabolite, the decrease in DHT, the active form of testosterone, has been proven to have a pro-arrhythmia effect by prolonging the QTc interval [52]. Testosterone decreases the L-type calcium current and increases certain potassium channel currents [52]. Consistent with our study, a lack of DHT may lead to a decrease in outward potassium channels that need to be verified. Additionally, DHA, one of the Omega-3 polyunsaturated fatty acids, may be protective against ventricular arrhythmia by inhibiting *I*_Na_ and *I*_Ca-L_ [53,54]. Further studies are needed to evaluate and explore the effects and mechanisms on cardiac electrophysiology.

In our study, we found the pro-arrhythmic effect of CUMS serum; however, the absence of pharmacological reversal represents a limitation. The CUMS serum reduced the repolarization of potassium currents by downregulating potassium channel expression. Notably, classical anti-arrhythmic drugs, which typically act by blocking receptors or ion channels, may lack specificity for the arrhythmias in our model. Thus, developing targeted strategies for the prevention and treatment of arrhythmias under chronic stress is warranted. Further studies are needed to identify critical substances and elucidate the molecular mechanisms underlying the reduction in ion channel proteins. This will facilitate the discovery of drugs that can restore potassium channel protein expression and achieve anti-arrhythmic effects in stress-induced arrhythmias.

In summary, our study first introduced a human cardiomyocyte model under stress by applying hiPSC-CMs incubated with a serum of a CUMS mice model. Humoral factors can induce the electrical remodeling of cardiomyocytes, and *I*_Ks_ is the most sensitive to the stimulation of humoral factors, displaying an obvious decrease. Metabolites in stress serum have the effect of inducing arrhythmia through chronic effects.

## 5. Conclusions

This study revealed the pro-arrhythmic effect and mechanism of chronic stress-associated humoral factors in hiPSC-CMs. We identified the decrease in currents of outward potassium ion channels associated with downregulated expression of functional proteins. And among the outward potassium ion channels we investigated, *I*_Ks_ showed the tendency to play the most important role. We accessed the changes in metabolites from chronic stress serum and validated their pro-arrhythmic effects. These findings may inspire new methods for the prevention of chronic stress-associated arrhythmia.

## Data Availability

The raw data supporting the conclusions of this article will be made available by the authors upon request.

## Supplementary Materials

The following supporting information can be downloaded at: https://www.mdpi.com/article/10.3390/biology14060652/s1, Figure S1: Body weight and behavioral changes in mice; Figure S2: Images of hiPSC-CMs; Figure S3: Acute effect of serum from mice with CUMS on repolarization currents; Figure S4: Original western blot figures for figure 4 (A,B,C); Table S1: Annotation of partially differential metabolites in the serum of CUMS mice.

## Abbreviations

The following abbreviations are used in this manuscript:

APD	Action potential duration
CUMS	Chronic unpredictable mild stress
hiPSC-CMs	Human-induced pluripotent stem cell-derived cardiomyocytes
EAD	Early afterdepolarization
ECG	Electrocardiogram
EFP	Extracellular field potential
EPM	Elevated plus-mazetest
FST	Forced swimming test
HEK	Human embryonic kidney
HPA	Hypothalamic–pituitary–adrenal axis
ICa-L	L-type calcium current
IKr	Rapid inward rectifier potassium current
IKs	Slow inward rectifier potassium current
iPSC	induced pluripotent stem cell
Ito	Electrocardiogram
ISO	Isoproterenol
OFT	Open field test
PCA	Principal component analysis
PVCs	Premature ventricular contractions
QTc	Corrected QT interval
SCD	Sudden cardiac death
SPT	Sucrose preference test
TST	Tail suspension test
VT	Ventricular tachycardia
