# Supplementary material for: Pro-Arrhythmic Effect of Chronic Stress-Associated Humoral Factors in Human Induced Pluripotent Stem Cell-Derived Cardiomyocytes
Source: Biology (Basel). 2025 Jun 4;14(6):652. doi: 10.3390/biology14060652 (PMC12189799; doi:10.3390/biology14060652)
Supplement: Supplementary file 1 [file biology-14-00652-s001.zip › biology-3648301-supplementary.pdf]

Proarrhythmic effect of chronic stress-associated humoral factors in human induced pluripotent stem cell-derived cardiomyocytes

Shi Su[1†], Jinglei Sun[1†], Suhua Qiu[1], Wenting Wu[1], Jiali Zhang[1], Yi Wang[1], Chenxia Shi[1], Yanfang Xu[1*]

1 Department of Pharmacology, Hebei Medical University; The Key Laboratory of New Drug Pharmacology and Toxicology, Hebei Province; The Key Laboratory of Neural and Vascular Biology, Ministry of Edu-cation, Shijiazhuang 050017, China; sushiwudi@163.com (S.S.); 18712919298@163.com (J.S.); qiusuhua1111@hotmail.com (S.Q.); 15903213515@163.com (W.W.); ll13931860169@outlook.com (J.Z.); btwy001@163.com (Y.W.); chenxiashi@163.com (C.S.); yanfangxu@hebmu.edu.cn (Y.X.)

† These authors contributed equally to this work.

* Correspondence: yanfangxu@hebmu.edu.cn; Tel.: 86-311-86266431

Supplementary Methods

Chronic unpredictable mild stress (CUMS) procedure

CUMS mice were chronically exposed to various stressors including water deprivation (24 h), food deprivation (24 h), cage tilting (45°, 24 h), wet bedding (24 h, 200 mL water per 100 g bedding), empty cage (24 h), tail clamping (1 cm from the tip of tail for 1 min), ice water swimming (5 min at 4 °C), inversion of the 12/12 h light/dark cycle for 24 h and restraint (24 h). Each animal was randomly subjected to two of these stressors each day, and the same stressor was not applied continuously so that mice could not predict the occurring stressor.

Behavioral tests

Behavioral tests were conducted after CUMS procedure. Anxiety-like and depression-like behavior were evaluated by open field test (OFT), elevated plus-maze (EPM) test, sucrose preference test (SPT), tail suspension test (TST), and forced swimming test (FST) respectively. OFT test was conducted in an experimental box (40 cm×40 cm×30 cm) with a camera on top. Mice were placed in the middle area of the floor and allowed to explore freely for 10 min. The floor was divided into 16 equal square grids and the track of the mice in the middle 4 grids were recorded for further comparison. EPM was performed with the apparatus of plus shape as previous research. Briefly, mice were placed in the cross site of the maze facing to the enclosed arm. The time in the open arm was recorded for following analysis. SPT test was conducted as previous study with a little adjustment. The mice were provided 2 bottles of 1% (w/v) sucrose for habituation on the 34th day of the experiment while water deprivation was performed on the last day. The mice were provided 1 bottle of 1(w/v) sucrose and 1 bottle of tap water for sucrose preference test for 24 h. Sucrose preference was calculated as follows: Preference (%) = Sucrose consumption/ (sucrose consumption + water consumption). TST was executed as reported previously. Mice were suspended by adhesive tape affixed on tails at 1 cm from the tip with 50 cm distance between head and floor. Total immobility time of the mice were recorded during a 6 min period. FST was carried out referred to the former study. Mice were place in the transparent cylindrical apparatus filled water (23-25°C) with the level 10 cm to the top. Immobility time was recorded during the 4 min test period after 2 min of adaption.

Patch-clamp recordings

For I_{to} and I_{Kr} recording, the external solution contained (in mM): NaCl 140, KCl 4, $MgCl_2$ 1, glucose 10, HEPES 10 (pH 7.4 with NaOH), and pipette solution contained

(in mM): KCl 140, $MgCl_2$ 1, Mg-ATP 4, EGTA 5, HEPES 10 (pH 7.2 with KOH). For I_{Ks} recording, the external solution contained (in mM): NaCl 140, KCl 5.4, $CaCl_2$ 2.0, MgCl2 1, Glucose 10, and HEPES 10 (pH 7.4 with NaOH) and the patch pipette solution contained (in mM) KCl 20, K-aspartate 110, HEPES 10, EGTA 5, Na_2-phosphocreatine 5, Mg-ATP, (pH 7.2 with KOH).

Immunofluorescence

The hiPSC-CMs were plated on confocal dishes. To identify cardiomyocytes, cells were cultured for at least 4 days before performing the immunofluorescence experiments. The cells were washed with PBS, fixed with 4% paraformaldehyde at 37 °C for 15 min, and permeabilized with 0.3% Triton X-100 and 3% BSA in PBS at 37 °C for 1 h. Then the cells were incubated with blocking solution (5% goat serum, 0.15% Triton X-100, and 1.5% BSA in PBS) for 30 min at 37 °C and incubated with primary antibodies at 4 °C overnight. The primary antibodies used were as follows: anti-cTnT antibody (Affinity, #DF6261) and anti-α-actinin antibody (Sigma, A7811), both diluted 1:200. Then, hiPSC-CMs were incubated with the secondary antibodies for 2 h at room temperature and protected from light. Cell nuclei were stained using Hoechst (Solarbio, Beijing, China). Secondary antibodies, including CoraLite 594-conjugated goat anti-mouse IgG (Proteintech) and CoraLite 488-conjugated goat anti-rabbit IgG (Proteintech), were diluted 1:500. Images were acquired on an Olympus two-photon laser scanning microscope. Olympus FV10-ASW-V4 software was used for image processing.

Supplementary Results

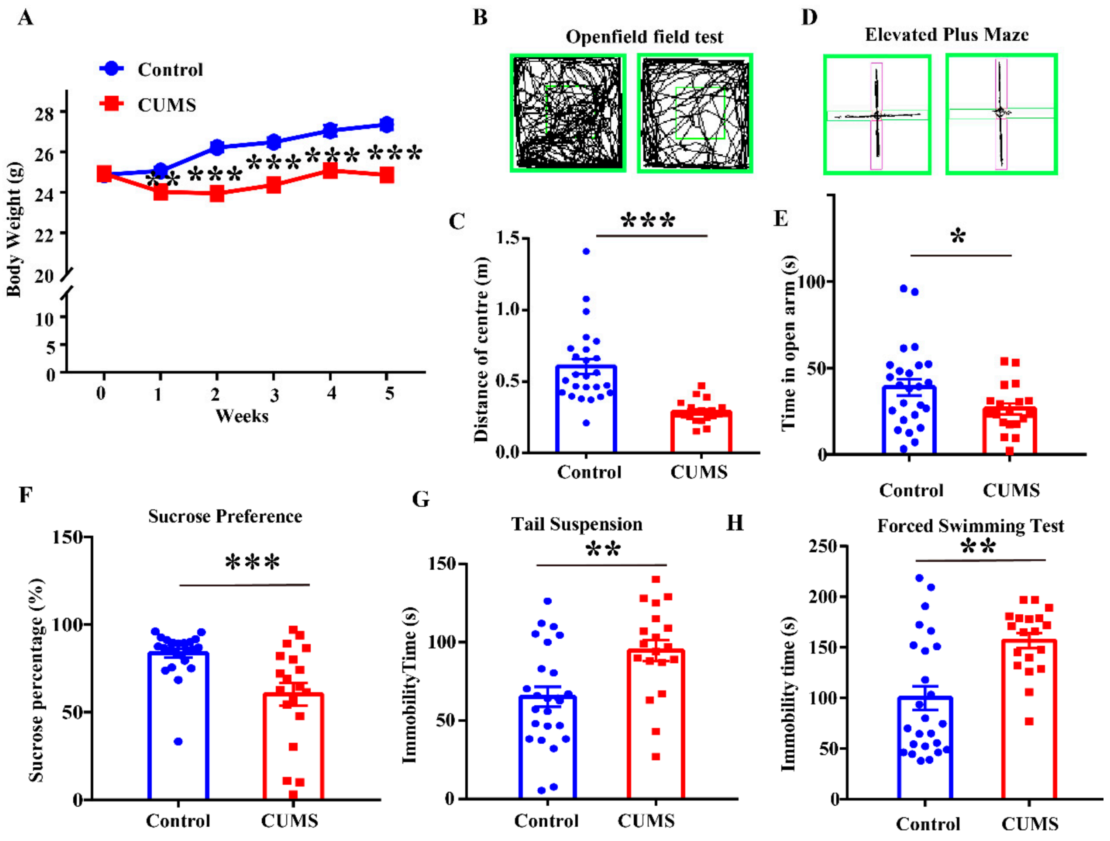

Figure S1 Body weight and behavioral changes in mice. (**A**) Body weight changes during the CUMS procedure. (**B-C**) Representative movement tracks and summary data of the open field test after CMS procedure. (**D-E**) Representative movement tracks and summary data of the elevated plus maze test after CMS procedure. (**F**) The sucrose preference in sucrose preference test. (**G**) The immobility time in tail suspension test. (**H**) The immobility time in forced swimming test. n = 25 in Control group, n = 19 in CUMS group. Mean ± SEM. *$p < 0.05$, **$p < 0.01$, ***$p < 0.001$ vs Control group.

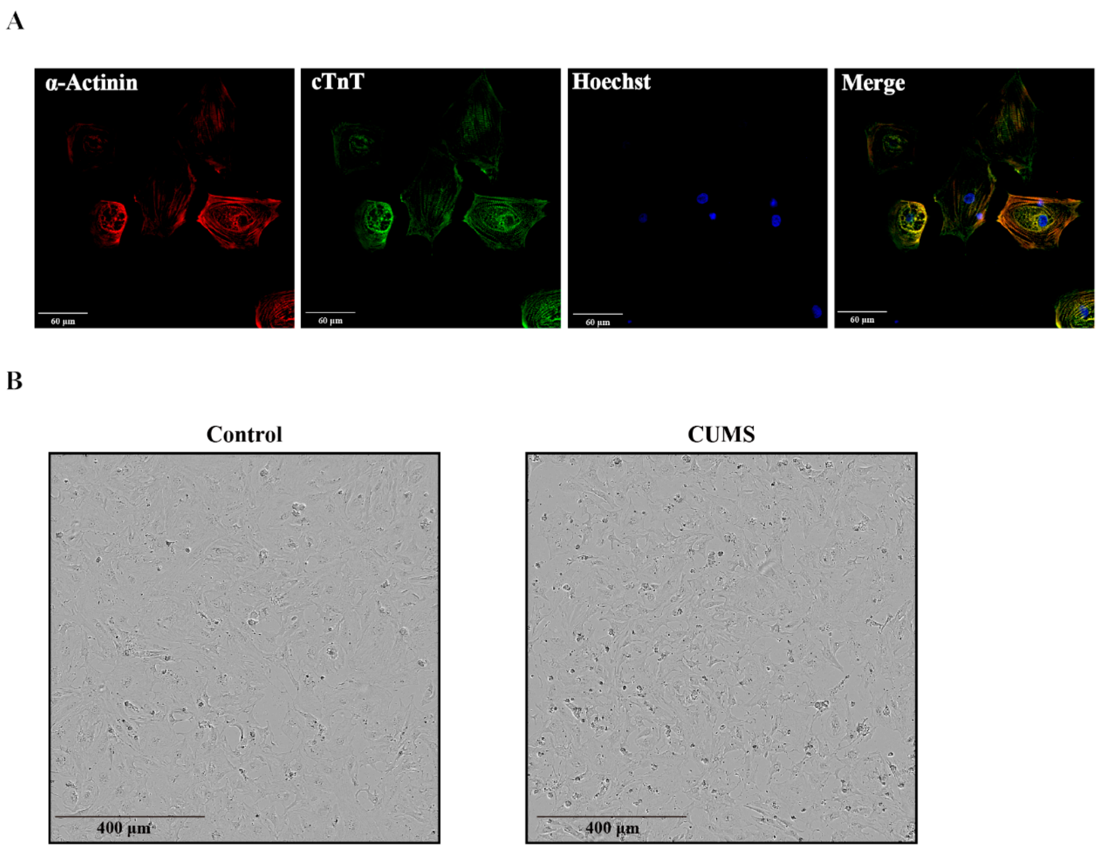

Figure S2 Images of hiPSC-CMs. (**A**) Identification of hiPSC-CMs. The hiPSC-CMs were immunostained with α-actinin antibody (red) and cTnT (green) antibody, with nuclei stained by Hoechst (blue). Scale bars: 60 μm. (**B**) Representative microscopic images of hiPSC-CMs treated with Control mouse serum or CUMS mouse serum for 48 h. Scale bars: 400 μm.

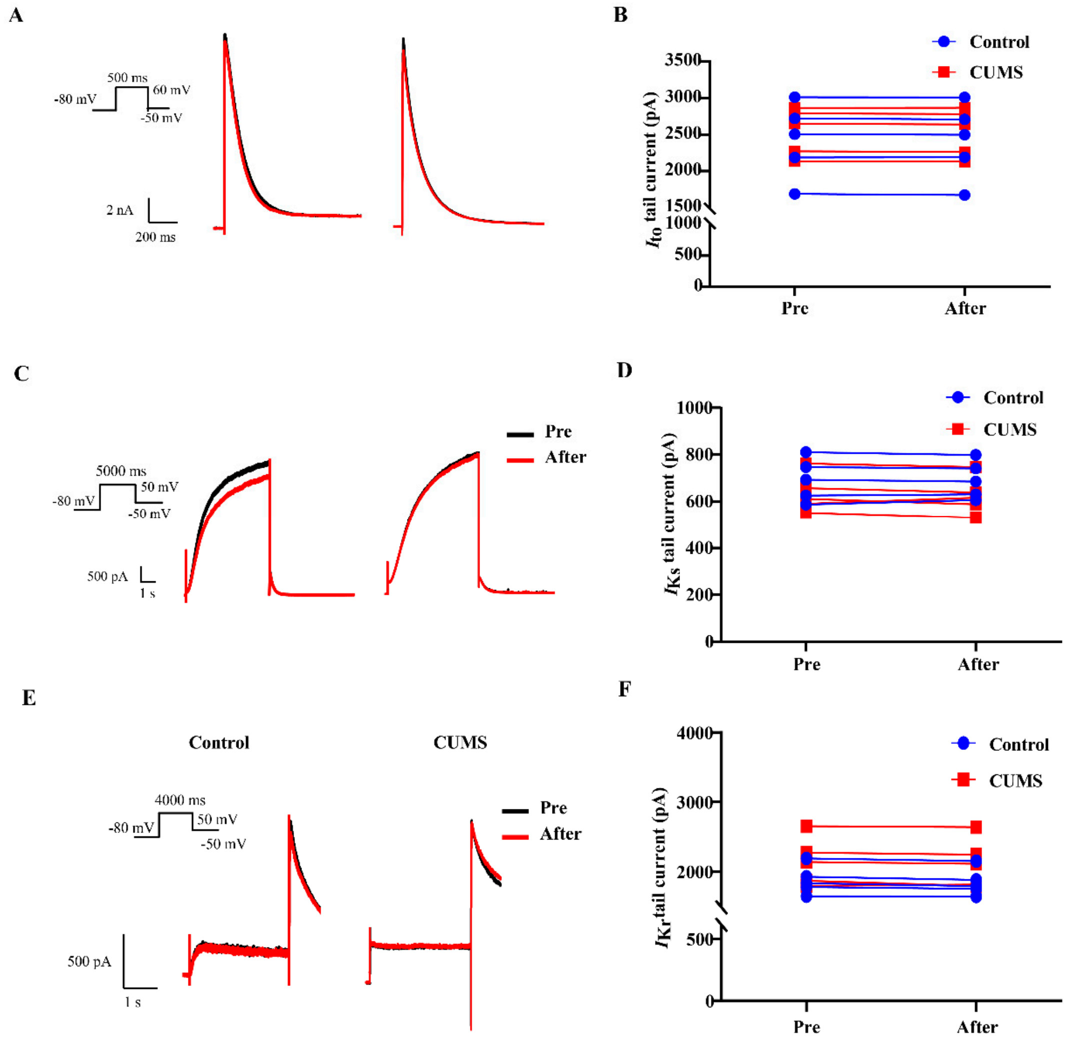

Figure S3 Acute effect of serum from mice with CUMS on repolarization currents. (**A**) Representative I_{to} traces recorded under the shown protocol in the presence of Control serum and CUMS serum for 5 min. (**B**) The pretreatement-after treatment scatter plot of current density change of I_{to}. n = 5. (**C**) Representative I_{Ks} traces recorded under the shown protocol in the presence of Control serum and CUMS serum for 5 min. (**D**) The pretreatement-after treatment scatter plot of tail current density change of I_{Ks}. n = 5. (**E**) Representative I_{Kr} traces recorded under the shown protocol in the presence of Control serum and CUMS serum for 5 min. (**F**) The pre-treatment and after treatment scatter plot of tail current density change of I_{Kr}. n = 5.

Table S1 Annotation of partially differential metabolites in the serum of CUMS mice

No.	Rt(min)	Ion mode	Compound	Formula	HMDB	MW	Trend
**1**	4.58	negative	Adenine	$C_5H_5N_5$	HMDB0000034	135.053	↑
**2**	3.486	positive	3-Dehydroxycarnitine	$C_7H_{15}NO_2$	HMDB0012154	145.1102	↓
**3**	3.311	positive	Adenosine	$C_{10}H_{13}N_5O_4$	HMDB0000050	267.0965	↑
**4**	3.805	negative	8-hydroxy-deoxyguanosine	$C_{10}H_{13}N_5O_5$	HMDB0003333	283.0915	↑
**5**	6.147	negative	Nonanoic acid	$C_9H_{18}O_2$	HMDB0000847	158.1293	↓
**6**	3.538	positive	Hypoxanthine	$C_5H_4N_4O$	HMDB0000157	136.0384	↑
**7**	4.291	positive	2-Hexenoylcarnitine	$C_{13}H_{23}NO_4$	HMDB0013161	257.1625	↓
**8**	3.501	positive	Cyclohexanecarboxylic acid	$C_7H_{12}O_2$	HMDB0031342	128.0837	↓
**9**	1.722	positive	Adenosine 5'-monophosphate	$C_{10}H_{14}N_5O_7P$	HMDB0000045	347.0627	↑
**10**	9.111	positive	(E)-2-decenoicacid	$C_{10}H_{18}O_2$	HMDB0010726	170.1304	↑
**11**	3.539	positive	Guanine	$C_5H_5N_5O$	HMDB0000132	151.0493	↑
**12**	3.564	positive	Guanosine	$C_{10}H_{13}N_5O_5$	HMDB0000133	283.0914	↑
**13**	8.66	positive	Methyl palmitate	$C_{17}H_{34}O_2$	HMDB0061859	287.282	↓
**14**	9.111	positive	3_6-Nonadienal	$C_9H_{14}O$	HMDB0031152	138.1043	↓
**15**	7.618	positive	Capryloylglycine	$C_{10}H_{19}NO_3$	HMDB0000832	201.1362	↓
**16**	8.742	negative	Prostaglandin H2	$C_{20}H_{32}O_5$	HMDB0001381	334.2144	↓
**17**	9.696	positive	Tetradecanedioicacid	$C_{14}H_{26}O_4$	HMDB0000872	258.1829	↓
**18**	8.401	negative	Leukotriene B4	$C_{20}H_{32}O_4$	HMDB0001085	336.2299	↓
**19**	9.894	positive	Undecanedioic acid	$C_{11}H_{20}O_4$	HMDB0000888	216.1359	↓
**20**	10.258	positive	Arachidonic acid	$C_{20}H_{32}O_2$	HMDB0001043	304.2398	↓
**21**	10.32	positive	Docosahexaenoicacid	$C_{22}H_{32}O_2$	HMDB0002183	328.2398	↓
**22**	10.16	positive	16-alpha-Hydroxypregnenolone	$C_{21}H_{32}O_3$	HMDB0000315	332.2347	↓
**23**	8.394	negative	(-)-Prostaglandin E1	$C_{20}H_{34}O_5$	HMDB0001442	354.2407	↓
**24**	10.467	negative	Methyl linoleate	$C_{19}H_{34}O_2$	HMDB0034381	294.2557	↓
**25**	10.048	negative	5α-Dihydrotestosterone	$C_{19}H_{30}O_2$	HMDB0002961	290.2243	↓
