# Supplementary figures and images for: Pro-Arrhythmic Effect of Chronic Stress-Associated Humoral Factors in Human Induced Pluripotent Stem Cell-Derived Cardiomyocytes
Source: Biology (Basel). 2025 Jun 4;14(6):652. doi: 10.3390/biology14060652 (PMC12189799; doi:10.3390/biology14060652)

Original WB data

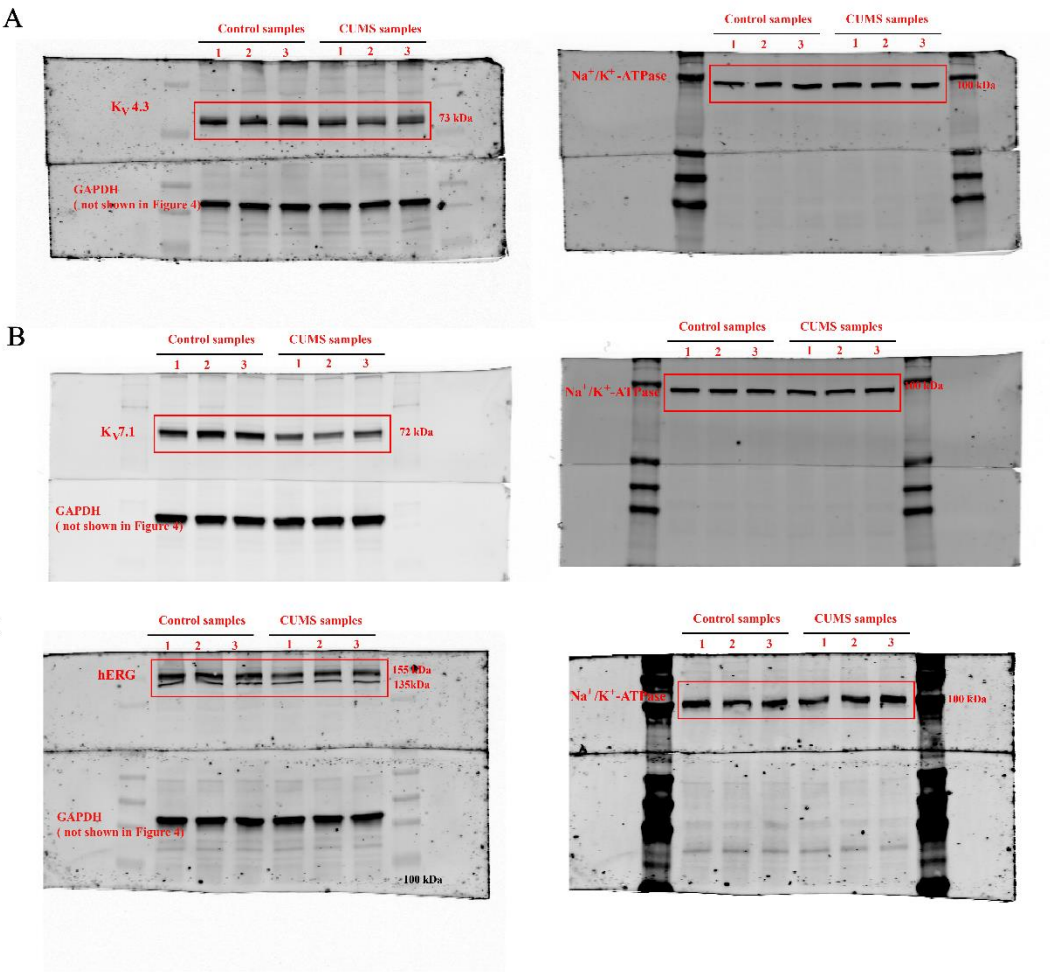

Supplement: Supplementary file 1 [file biology-14-00652-s001.zip › biology-3648301-figure S4.pdf]
